# Experimental Approaches and Computational Modeling of Rat Serum Albumin and Its Interaction with Piperine

**DOI:** 10.3390/ijms20122856

**Published:** 2019-06-12

**Authors:** Gabriel Zazeri, Ana Paula Ribeiro Povinelli, Marcelo de Freitas Lima, Marinônio Lopes Cornélio

**Affiliations:** 1 Departamento de Física, Instituto de Biociências, Letras e Ciências Exatas (IBILCE), UNESP, Rua Cristovão Colombo 2265, São José do Rio Preto CEP 15054-000, SP, Brazil; gabriel.zazeri@unesp.br (G.Z.); ana.povinelli@unesp.br (A.P.R.P.); 2 Departamento de Química, Instituto de Biociências, Letras e Ciências Exatas (IBILCE), UNESP, Rua Cristovão Colombo 2265, São José do Rio Preto CEP 15054-000, SP, Brazil; marcelo.f.lima@unesp.br

**Keywords:** piperine, rat serum albumin, fluorescence spectroscopy, comparative modeling, molecular docking, molecular dynamics

## Abstract

The bioactive piperine (1-piperoyl piperidine) compound found in some pepper species (*Piper nigrum linn* and *Piper sarmentosum Roxb*) has been shown to have therapeutic properties and to be useful for well-being. The tests used to validate these properties were performed in vitro or with small rats. However, in all these assays, the molecular approach was absent. Although the first therapeutic trials relied on the use of rats, no proposal was mentioned either experimentally or computationally at the molecular level regarding the interaction between piperine and rat serum albumin (RSA). In the present study, several spectroscopic techniques were employed to characterize rat serum albumin and, aided by computational techniques, the protein modeling was proposed. From the spectroscopic results, it was possible to estimate the binding constant (3.9 × 10^4^ M^−1^ at 288 K) using the Stern–Volmer model and the number of ligands (three) associated with the protein applying interaction density function model. The Gibbs free energy, an important thermodynamic parameter, was determined (−25 kJ/mol), indicating that the interaction was spontaneous. This important set of experimental results served to parameterize the computational simulations. The results of molecular docking and molecular dynamics matched appropriately made it possible to have detailed microenvironments of RSA accessed by piperine.

## 1. Introduction

Piperine (1-piperoyl piperidine), whose molecular weight corresponds to 285 g/mol, is an alkaloid present in many types of plants, such as *Piper nigrum linn* (black pepper), *Zingiber officinale* (ginger), *Piper longum L.*, *Piper sarmentosum Roxb,* and *Dangzuo* [1,2,3,4,5,6]. Piperine is not only used as a seasoning but also in various preparations of traditional medicine in India; it is also employed in the oldest medical science practiced since ancient time (Ayurveda) [7]. It presents a series of important pharmacological actions, such as anti-inflammatory [8,9,10,11], anti-carcinogenic [12,13,14,15,16], antimicrobial [17,18,19], and antiparasitic [20,21]. Besides that, piperine plays a crucial role in enhancing the bioavailability of several drugs by inhibiting the drug metabolizing enzymes, which retards the clearance of those compounds [22].

Studies in vivo with rats reported in the literature [23] showed that after 6 h of oral administration, piperine presented the highest concentration in serum as well as in several organs, and its presence remained up to 96 h after its administration and was then excreted from the organism. As piperine is a lipophilic molecule (LogP = 3.69) [24], its transport through the plasma depends on proteins capable of carrying lipophilic molecules, such as albumin. Although studies have shown the pharmacokinetics of piperine in rats, the molecular mechanism involved in the interaction of piperine toward rat serum albumin (RSA) is still unknown. There is interest from the scientific community in the plasticity of albumin, since it can transport several different exogenous ligands [25]. This protein is synthesized in the liver and remains soluble in plasma [25]. Rat serum albumin (RSA) contains 584 amino acids and just one tryptophan residue at position 214 [26], which allows the use of fluorescence spectroscopy to evaluate the interaction of small molecules with it. Various albumin structures of different species are available on the Protein Data Bank (PDB) and, according to structural characterization, serum albumins are composed mostly by α-helix and contain disulfide bridges [25], which confer to the protein a melting temperature of ~331 K [27].

The lack of information on the tertiary structure of RSA together with the absence of multispectroscopic data analyses and the complete unavailability of computer simulation data were sufficient reasons to carry out this study.

The present work aims to fill this information gap with respect to RSA using several experimental techniques with the help of computational simulations to elucidate models of the interaction of piperine into RSA. The interaction density function (IDF) method was applied to the fluorescence spectroscopy data in order to determine the binding constant, since the value of the binding constant is essential to comprehend the affinity of piperine toward RSA and therefore the efficiency of its transport in the blood stream. A moderate binding constant (10^4^–10^6^ M^–1^) is desirable because it reflects an extended half-life and a better distribution of the compound in the body [28]. With the IDF method, it was possible to determine the number of sites and the existence (or otherwise) of cooperative properties between them.

Circular dichroism (CD) spectroscopy was applied to determine the RSA secondary structure fractions and to elucidate if there were changes in the secondary structure of RSA under the effect of temperature, in the presence of methanol, or due to interaction with piperine. A model for RSA structure was built using comparative modeling, which allowed the use of molecular docking to find the interaction sites of piperine on RSA. The complex stability was verified with molecular dynamics simulations.

## 2. Results and Discussion

### 2.1. Fluorescence Spectroscopy

**Figure 1:**
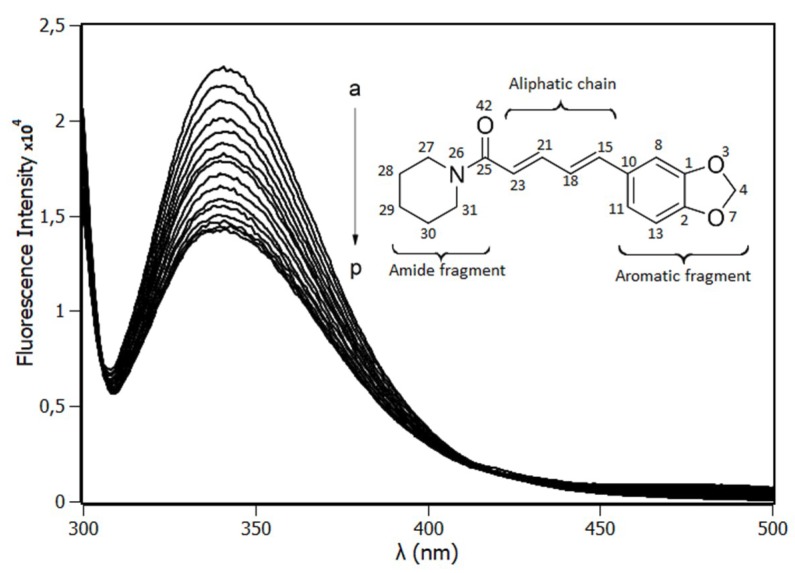
Rat serum albumin (RSA) emission spectra from 305 to 500 nm [RSA] = 4.0 μM. Piperine concentration from 0 to 15 μM (a→p) with increments of 1 μM (pH 7.4, T = 288 K, λ_exc_ = 295 nm). The insert is the molecular structure of piperine.

Figure 1 shows the fluorescence emission spectra of RSA in the absence (a) and the presence (→ p) of piperine. The interaction of RSA with piperine was monitored by following the intensity of rat serum albumin emission spectra at 340 nm. According to the spectra, the intensity of the RSA fluorescence signal decreased with the concentration increment of piperine, demonstrating the existence of the quenching effect upon the Trp214 emission.

The quenching mechanism can be classified either dynamic (diffusive encounters) or static (non-radiative complex formation) processes. It was possible to differentiate them by analyzing the dependence of Stern–Volmer constant (K_SV_) with temperature (Equation (1)) [29].

(1) $$\frac{F_{0}}{F}=1+K_{SV}\cdot \left[piperine\right]$$

**Figure 2:**
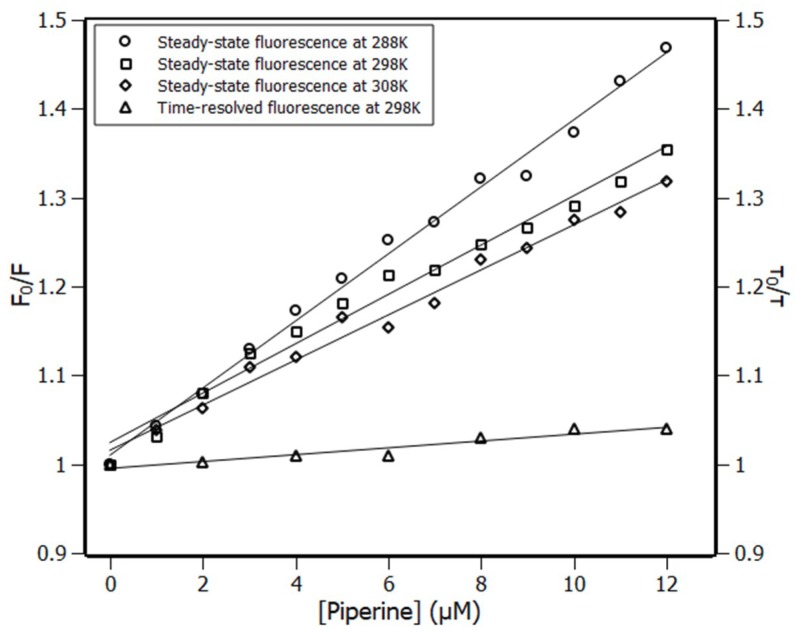
Left ordinate Stern–Volmer plots at three temperatures, 288 K, 298 K, and 308 K, and right ordinate time-resolved fluorescence lifetime plot at 298 K; [RSA] = 4 μM, [piperine] = 0–12 μM.

The Stern–Volmer plots (Figure 2) presented a linear response to the increment of piperine concentration. At temperatures 288 K, 298 K, and 308 K, the K_SV_ constant showed a decrement noticeable by the drop in slope of the linear adjustments, which was evidence of the static quenching process [30]. This meant that the endogenous fluorophore (Trp214) and the quencher (piperine) must have formed a non-fluorescent complex while interacting with each other [29].

The quenching mechanism obtained by steady-state fluorescence was checked by time-resolved experiments. In this experiment, the RSA tryptophan lifetimes of excited states were measured in the absence (τ_0_) and the presence (τ) of different concentrations of piperine. The ratio (τ_0_/τ) of their fluorescence lifetime was plotted at the right ordinate (Figure 2). Figure 2 showed that the ratio τ_0_/τ remained closed to the unity, which indicated that piperine poorly affected the RSA tryptophan fluorescence lifetime and confirmed that the quenching mechanism was static, which was in agreement with the steady state fluorescence measurements [29]. Such results once again indicated that piperine was on the outskirts of the Trp214 residue.

**Figure 3:**
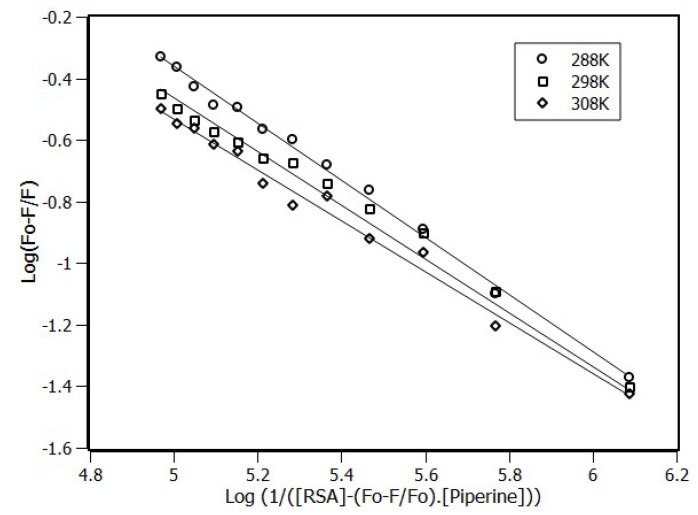
Double-log plots for the fluorescence quenching of RSA (4 μM) by the presence of piperine at 288 K, 298 K, and 308 K.

The double logarithmic approach, which is based on the chemical equilibrium reaction, is widely used to determine the binding constant (K_a_) and the number of sites (n) accessed by the ligand (Equation (2)) [31]. The values of K_SV_, K_a_, and n obtained by linear regression (Figure 3) are summarized in Table 1.

(2) $$\log\left(\frac{F_{0}-F}{F}\right)=n\cdot \log K_{a}-n\cdot \log\left(\frac{1}{\left[piperine\right]-\left(\frac{F_{0}-F}{F}\right)\cdot \left[RSA\right]}\right)$$

The K_a_ for the three temperatures remained close to the order of magnitude of 10^4^ M^−1^. According to the literature, this meant that the binding constant was moderate for the albumin proteins from different species [32,33,34]. The number of sites, n, remained close to the unity, which was in concordance with the first order binding equilibrium model.

The stability of the complex was slightly affected by temperature changes, since K_a_ and K_SV_ decreased with temperature increment. Such a response was understandable considering that the same physical observable was used in both calculations, i.e., the variation in the fluorescence signal intensity.

### 2.2. Thermodynamic Parameters

An important source of information came from both constants, i.e., the thermodynamic parameters, which unveiled the thermodynamic balance of the ligand protein interaction. This information was able to describe which physical forces influenced the formation of the complex [35]. Entropy variation (∆S) and enthalpy variation (∆H) were determined according to the Van’t Hoff equation (Equation (3)) followed by the Gibbs free energy variation (Equation (4)).

(3) $$\ln K_{a}=-\frac{∆H}{R\cdot T}+\frac{∆S}{R}$$

where *K_a_* is the binding constant calculated by the binding equilibrium model, *R* is the ideal gas constant, and *T* is the system temperature.

**Figure 4:**
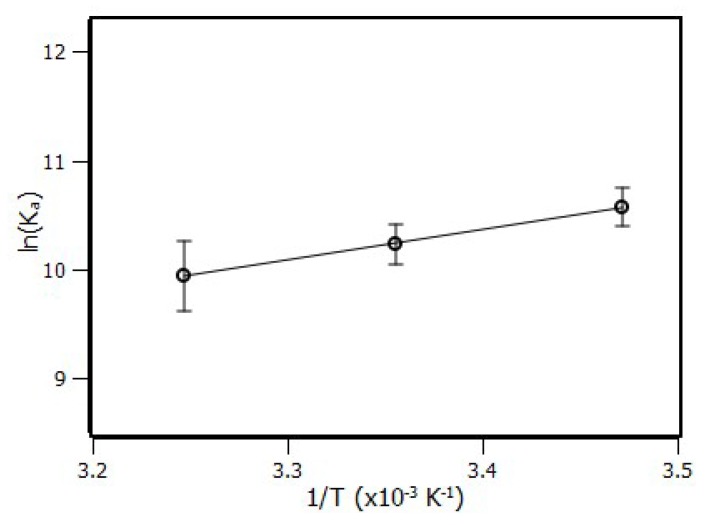
Van´t Hoff plot for the complex RSA–piperine at 288 K, 298 K, and 308 K.

The results from Figure 4 are summarized in Table 2, showing that temperature did not have influence on ∆G values due to the low contribution of the entropic term. On the other hand, the negative values of ∆G indicated that the complex formation was spontaneous.

(4) ∆G=∆H−T∆S

By comparing the values obtained between enthalpy and entropy, it could be seen that the enthalpy contribution was more significant. This showed that interactions of the type hydrogen bonds and the Van der Waals force were most likely playing the main role in the stabilization of the complex [34,35,36,37].

### 2.3. Interaction Density Function (IDF)

An alternative methodology was employed to either support the treatment of the data using the binding equilibrium model with the Stern–Volmer approach or to better elucidate the protein binding interaction. In principle, this methodology considers two important factors, the average interaction density (Σν_i_) and the free ligand concentration ([piperine]_Free_). In essence, the interaction density function treats the relation between them using equilibrium binding isotherms [38]. Making use of the same physical observable (fluorescence signal) but in a percentage mode, the fluorescence quenching percentage (ΔF) is given by Equation (5).

(5) $$∆F=\frac{\left|F-F_{0}\right|}{F_{0}}\;\cdot \;100\%$$

where *F*_0_ is the observed fluorescence signal in the absence and *F* in the presence of piperine.

**Figure 5:**
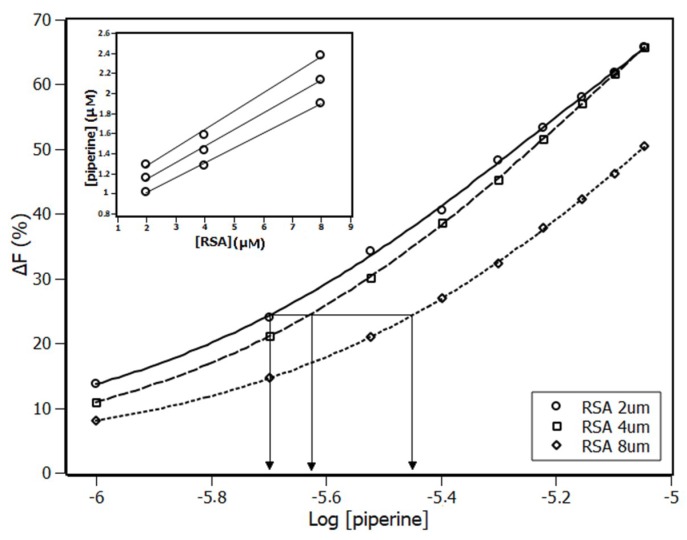
Plot of fluorescence titration of piperine with RSA at three different protein concentrations (2 μM, 4 μM, and 8 μM) at 288 K. Insert plot contains three sets of concentration pairs ([piperine]:[RSA]).

In Figure 5, the sigmoid-type curves show that, at the same value of ∆F, the concentrations of free ligand ([piperine]_Free_) and the average ligand interaction density distributions (Σν_i_) are the same, regardless of the total protein concentration [RSA]. The relationships built with these variables obey the mass conservation equation (Equation (6)).

(6) $$\left[piperine\right]={\left[piperine\right]}_{free}+\left(\sum \nu_{i}\right)\cdot \left[RSA\right]$$

The insert in Figure 5 shows the plot [piperine] versus [RSA], where [piperine]_Free_ is the y-intercept and ∑ν_i_ is the slope.

**Figure 6:**
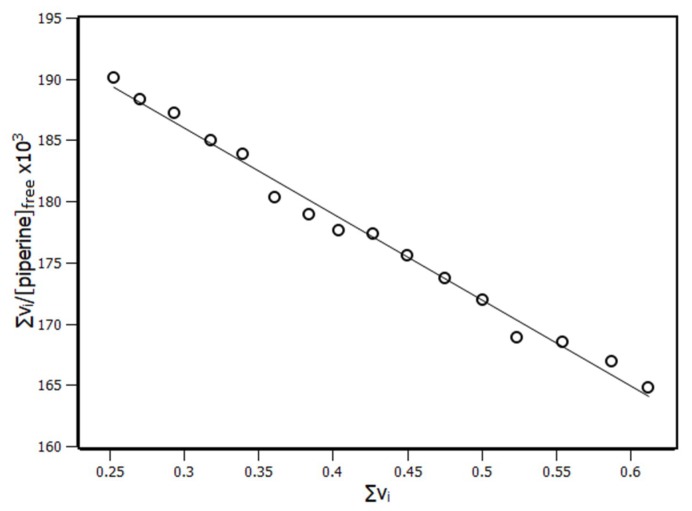
Scatchard plot of RSA and piperine at 288 K based on the interaction density function (IDF) model.

According to the IDF results, a Scatchard plot was built (Figure 6). This plot presented an unique linear profile, which meant that all RSA binding sites were equivalent and independent, exhibiting a non-cooperative state [39]. The number of sites and the binding constant (K_b_) were obtained through linear regression based on Equation (7).

(7) $$\frac{\sum \nu_{i}}{{\left[piperine\right]}_{free}}=n\cdot K_{b}-\;K_{b}\cdot \sum \nu_{i}$$

The IDF method with the Scatchard plot revealed three binding sites with binding constant K_b_ of 7 × 10^4^ M^−1^. Such methods (binding equilibrium model and IDF) showed that, although the results obtained were in the same order of magnitude for the binding constant 10^4^ M^−1^, the same could not be said about the number of sites. Such results showed that both methods had reasonable agreement, revealing that the complex had an affinity constant that relayed between 10^4^–10^6^ M^−1^, which was a moderate constant for most of the albumin proteins [32,33,34]. Although the binding constant (K_b_) obtained from the Scatchard plot had the same order of magnitude of K_a_ obtained from the double log plot, the difference between the number of binding sites indicated that the IDF method was more realistic.

### 2.4. Circular Dichroism Spectroscopy

**Figure 7:**
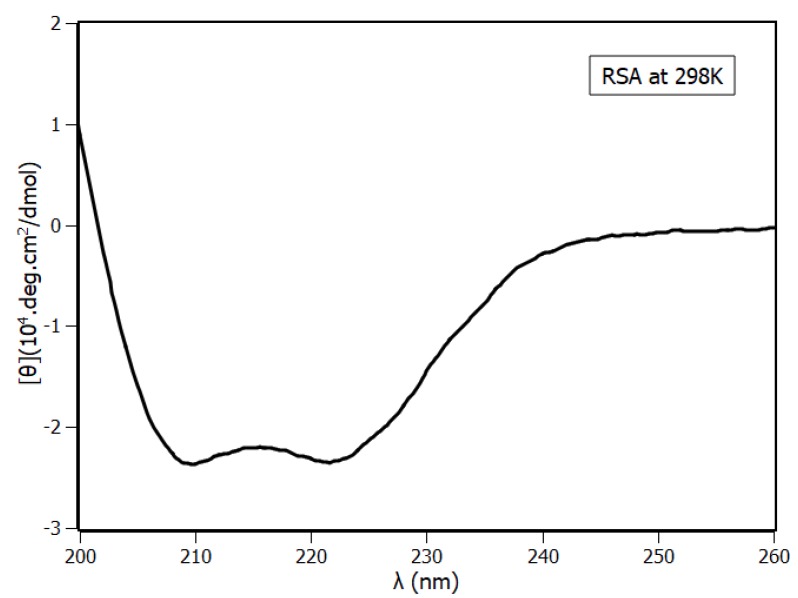
Circular dichroism of RSA at pH 7.4 and 298 K, [RSA] = 4 µM.

A recurring issue in relation to the protein binding interaction is whether the presence of the ligand alters protein conformation. To elucidate this question and obtain support information to validate the RSA structure model, circular dichroism (CD) spectroscopy was used. The CD spectrum of RSA (Figure 7) presented negative bands at 208 nm (π-π*) and 222 nm (n-π*), which were characteristics of α-helix content [40,41]. According to spectrum analyses, RSA was composed mainly of α-helix with 63%, turns with 17%, and random coil with 17%. Tests of RSA secondary structure stability were performed in five different conditions—at the highest concentration of piperine, at the highest concentration of methanol, and at three temperatures (288 K, 298 K, and 308 K). Results indicated that RSA secondary structures were preserved in all the conditions (Figures S3 and S4 and Table S2).

### 2.5. Protein Comparative Modeling

**Figure 8:**
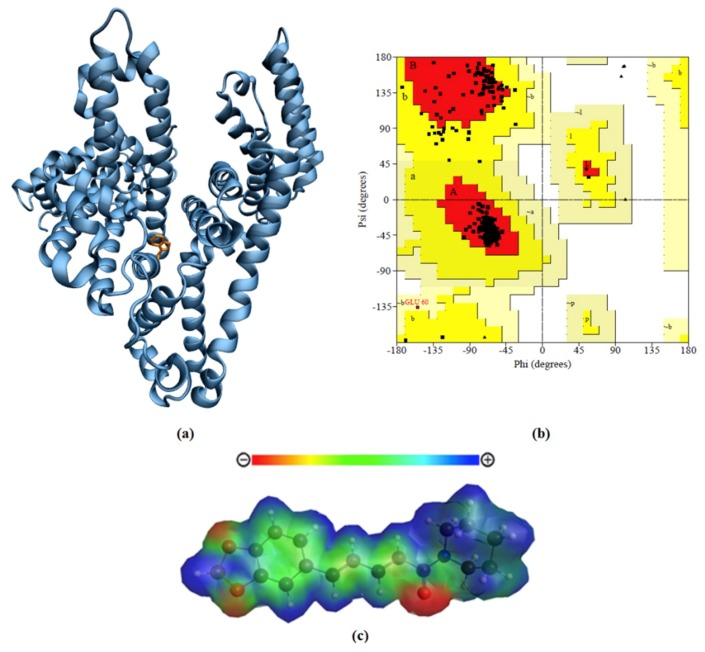
(**a**) RSA structure by comparative modeling with highlight on tryptophan in orange, (**b**) Ramachandran’s plot for RSA model, and (**c**) piperine electrostatic potential map (MEP).

Figure 8a shows the comparative model for RSA built by MODELLER using equine serum albumin (5HOZ) as a template, which had 73% similarity with RSA (Figure S5). According to the Ramachandran plot (Figure 8b), the model for RSA had a good stereochemical quality, since 95% of the dihedral angles (φ and ψ) were in the most favored regions. There were only three glycines (triangles) in the unfavorable area without compromising the stereochemical quality of the model, insofar as the glycine residue did not have the side chain and therefore was less restricted.

The percentage of the secondary structure of the model was calculated through visual molecular dynamics software (VMD) (Table 3). Based on the analysis, the RSA structure obtained by comparative modeling was composed mostly of α-helix with 75%, followed by 11% of turns and 11% random coil. Employing a molecular dynamics simulation during 50 ns, the structure presented 65% of α-helix, 17% of turns, and 16% of random coil. Note that the structure obtained after 50 ns of simulation had a composition of secondary structures close to that obtained by circular dichroism. This result showed that 50 ns was important to the relaxation of the protein.

### 2.6. Ab Initio Parametrization of Piperine

Piperine structure optimization by ab initio calculations presented a planar structure without torsional freedom for the whole structure except for a little flexibility of the amide bond (see the PDBQT file in the Supplementary Materials). This result was in agreement with the resonance of the conjugated second order bonds. Furthermore, no imaginary frequencies were found, which indicated that the structure found was not a transition state structure.

The theoretical Raman spectrum of piperine (Figure S6) presented the same pattern of the spectrum obtained experimentally [42]. According to experimental results reported in literature, piperine exhibited characteristics peaks in the range between 1100 and 1670 cm^−1^. The peaks found between 1580 and 1700 cm^−1^ were assigned to the aromatic and aliphatic –C=C– and –N–C=O stretching vibrations. The peaks at 1448 cm^−1^, 1153 cm^−1^, and 1295 cm^−1^ were assigned to –CH_2_ bending, –C–C– stretching, and –CH_2_ twisting vibrations, respectively [42]. The agreement between the theoretical and the experimental Raman spectra indicated that the piperine frame was very realistic about the true structure. Moreover, piperine electrostatic potential map (MEP) (Figure 8c) showed negative charge density around oxygen atoms, positive charge density in the molecule extremities, and a neutral area in the intermediate region.

### 2.7. Molecular Docking

**Figure 9:**
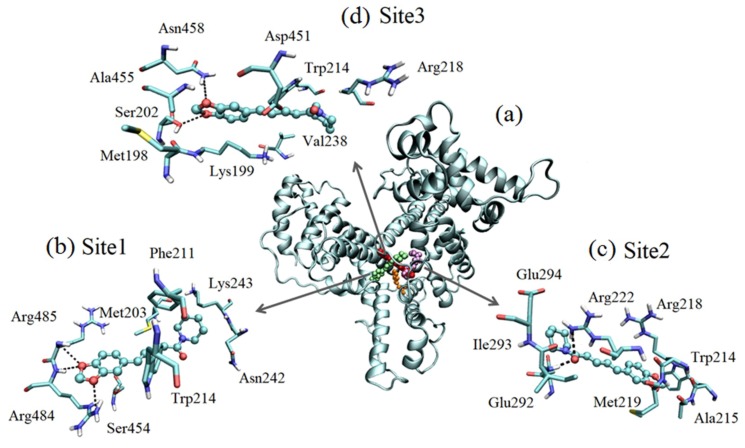
(**a**) The central picture represents the molecular docking. In orange is Trp214. The piperine molecules are in green (site 1), in purple (site 2), and in red (site 3). Frame (**b**) represents site 1, (**c**) site 2, and (**d**) site 3, with dotted lines indicating the hydrogen bonds.

Three promising sites were found by molecular docking (Figure 9) nominated as sites 1, 2, and 3, whose energy score conformation values were −6.7, −6.6, and −6.4 kcal/mol, respectively. In all configurations, piperine was close to Trp214, and such a position reinforced the results presented in Section 2.1 due to the quenching effect, which occurred in the presence of piperine. Considering the results of the energy score had very close values, this suggested that there was probably no distinction of access to sites by piperine. The binding constants determined by K_b_ = e^(−ΔG/RT)^ were 7.5 × 10^4^ M^−1^, 6.4 × 10^4^ M^−1^, and 4.6 × 10^4^ M^−1^ for sites 1, 2, and 3, respectively. The magnitudes of the values of each binding constant were similar to those found experimentally with the binding equilibrium model (3.9 × 10^4^ M^−1^) and the IDF method with the Scatchard plot (7 × 10^4^ M^−1^).

It is important and necessary to emphasize those three experimental results that supported the computational simulations, which were the number of binding sites, the thermodynamic parameters, and the proximity between piperine to the endogenous protein marker (Trp214). Therefore, the computational simulations were parameterized by the experimental findings and added detail to the participation of the residues at each binding site. The information contained in Table 2 shows that the Gibbs free energy was negative, thus the process was spontaneous for all sites provided by RSA. Besides that, the enthalpic–entropic balance showed that the greatest contribution came from enthalpy. Consequently, it was expected that more targeted interactions would occur (such as hydrogen bonds) without neglecting non-specific interactions such as Coulomb potentials, van der Waals, and hydrophobic interactions composing the entropic contribution.

At site 1 with an energy score of −6.7 kcal/mol, piperine was interacting with the side chain of polar amino acids such as Asn242 and Ser454. Piperine negatively charged the region formed by oxygen atoms O3 and O7 and interacted with positively charged amino acids, Arg484 and Arg485 (at pH 7.4) (Figure 8c). Furthermore, the arginines were performing three hydrogen bonds with piperine, two of them with atom O7 with lengths of 2.83 Å and 3.17 Å, and one with O3 with a length of 2.97 Å. Piperine was also interacting with the side chain of apolar amino acids such as Phe211, Trp214, and Met203, which were involved in hydrophobic contacts with the atoms of carbons of the aliphatic chain of the piperine (C15, C18, C21, and C23).

At site 2 with an energy score of −6.6 kcal/mol, piperine was interacting with Arg218 and Ile293 and performing two hydrogen bonds with just one oxygen atom at the amide fragment (O42) with lengths of 3.13 Å and 2.91 Å, respectively. Arg218 and Arg222, which are positively charged amino acids (pH 7.4), were interacting with oxygen atoms of the aromatic fragments O3 and O7. The negatively charged amino acids Glu292 and Glu294 (pH 7.4) were interacting with the positively charged region of piperine, the amide fragment (C27, C28, C29, C30, and C31), according to MEP (Figure 8c). Met219 and Ala215 were involved in hydrophobic contacts with the aliphatic region of piperine.

At site 3 with an energy score of −6.4kcal/mol, piperine was interacting with Asn458 and Ser202 and performing two hydrogen bonds with piperine oxygens of the aromatic fragment with lengths of 3.1 Å (O3) and 2.77 Å (O7), respectively. The polar amino acids Ser202 and Asp451 were interacting with piperine polar aromatic fragments. The positively charged amino acid Arg218 was interacting with the oxygen atom O42 of the amide fragment; at the other extremity, the positively charged amino acid Lys199 was interacting with oxygens O3 and O7 of the aromatic fragment. The hydrophobic amino acids Ala455, Met198, and Val238 were involved in contacts with carbons of the aliphatic chain (C15, C8, C21, and C23) and the amide fragment (C27, C28, C29, C30, and C31).

The presence of hydrogen bonds in each site that contained polar residues was consistent with the enthalpic character of the interactions determined experimentally (see Section 2.2) without neglecting the participation of residues whose characteristics pointed to the entropic contribution, even in smaller quantities.

### 2.8. Molecular Dynamics Simulations

**Figure 10:**
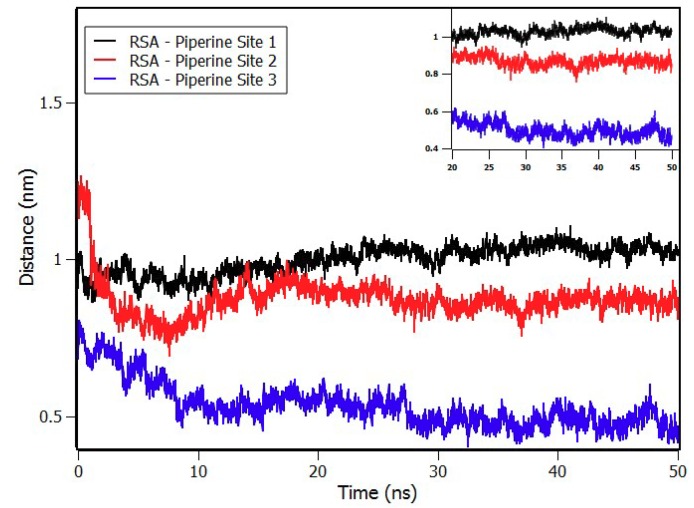
Distances between the center of geometry of RSA to the center of geometry of piperine at site 1 (black), at site 2 (red), and at site 3 (blue) during 50 ns of simulation.

The temporal stability of the protein was checked by the Root Mean Square Deviation (RMSD) and radius of gyration (R_g_) (Figures S7–S15). Both demonstrated small fluctuations after 20 ns of simulation, showing that the protein remained stable. A second test consisted of calculating the distance between the center of geometry (COG) of piperine to the center of geometry of RSA (Figure 10), which showed that after 20 ns of simulation, the distance remained stable, suggesting that the complex was stabilized.

**Figure 11:**
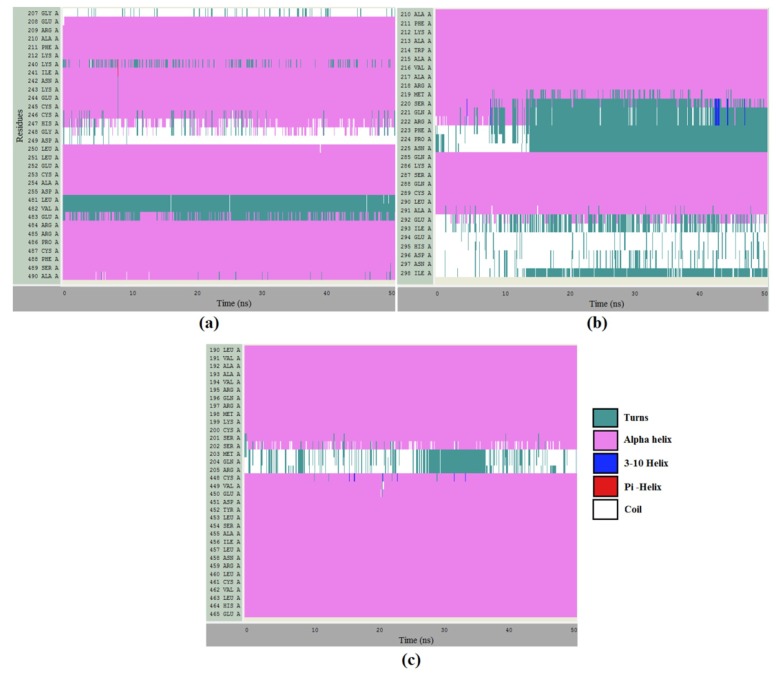
Molecular dynamic simulation along 50 ns of site 1 (**a**), site 2 (**b**), and site 3 (**c**), showing the smooth running of the α-helix content.

The stability of segments of RSA that were involved in the interaction with piperine was verified. Figure 11a shows the stability of the secondary structures presented on site 1 α-helix (pink color). Isolated fluctuations were observed during the 50 ns as Lys240, Cys246, and Glu483 changed from α-helix to turn and His247 changed from α-helix to coil. At site 2 (Figure 11b), during the first 10 ns, the amino acids Ser220, Gln221, and Arg222 (which were at the N-terminal of the α-helix) became part of a turn, remaining in this state until the end of the simulation. Moreover, the segment of amino acids from 292 to 298 fluctuated between random coil and turns.

A similar response was found for site 3 (Figure 11c). According to the results, only three amino acids (Met203, Gln204, and Arg205) did not compose the α-helix content. During the 50 ns, all amino acids in the α-helix conformation kept the structure except for Ser202, which presented a few fluctuations. The evidence collected by the simulations indicated that the protein had both local and global stability guaranteed, regardless of the presence or the absence of piperine.

## 3. Materials and Methods

### 3.1. Reagents

Piperine (> 97%) and rat serum albumin (> 99%) were purchased from Sigma-Aldrich Chemical Co. (Schnelldorf, Bavaria, Germany), as was dibasic sodium phosphate (> 99%) reagents, anhydrous citric acid (> 99%), and sodium chloride (> 99%). Methanol alcohol was purchased from Dynamics Química Contemporânea LTDA (Indaiatuba, SP, Brazil). All the materials purchased were used as supplied. Ultrapure water was prepared by a Millipore water purification system -Direct-Q UV-3(Merck KGaA, Darmstadt, Germany). Lyophilized rat serum albumin was reconstituted in 50 mM phosphate buffer containing 150 mM sodium chloride, and the pH was adjusted to 7.4 with anhydrous citric acid. Stock solutions of piperine were prepared in pure methanol. The concentrations of piperine and RSA solutions were determined by UV-VIS experiments performed on Biospectro spectrophotometer (Biospectro, Curitiba, PR, Brazil), using the extinction coefficient at 16,500 M^−1^cm^−1^ at 345 nm for piperine and 38,915 M^−1^cm^−1^ at 280 nm for RSA.

### 3.2. Steady-State Fluorescence Spectroscopy

Fluorescence experiments were performed on the Lumina (Thermo Fisher Scientific, Waltham, MA, USA) stationary state spectrofluorimeter equipped with thermal bath and Xenon lamp. A 3 mL quartz cuvette with a 10 mm optical path was used in the experiments. The widths of the excitation and the emission slits were adjusted to 10 nm. The wavelength of 295 nm was used to excite the single tryptophan residue of RSA (Trp214). The emission spectra were obtained in the range from 305 to 500 nm with a resolution of 1.0 nm ± 5.0 nm. Each emission point collected was the average of 15 accumulations. The software ScanWave was used to collect the measured data.

In the binding equilibrium experiments, small aliquots of piperine (increment of 1 μM) were added in RSA solution at 4 μM. Measurements were performed at 288 K, 298 K, and 308 K. In the interaction density function analysis, small aliquots of piperine (increments of 1 μM) were added in RSA solutions at 2 μM, 4 μM, and 8 μM at a fixed temperature (288 K). In all experiments, the final volume of methanol in the buffer was less than 1.2%. The influence of methanol in the RSA fluorescence emission was verified by adding small aliquots (increment of 1 μL) in 4.0 μM of RSA solution (Figure S1)

The correction of the inner filter effects was done with Equation (8), where F_corr_ and F_obs_ are corrected and observed fluorescence intensities, and A_ex_ and A_em_ are the absorbance at the excitation and the emission wavelengths, respectively [29].

(8) $$F_{corr}=F_{obs}\;{\cdot 10}^{\frac{\left(A_{ex}+A_{em}\right)}{2}}$$

### 3.3. Time-Resolved Fluorescence

Fluorescence lifetime measurements were performed using a Mini-tau filter-based fluorescence lifetime spectrometer coupled to a Time-Correlated Single Photon Counting (TCSPC) system (Edinburgh Instruments, Livingston, UK). Aliquots of piperine were added in the RSA solution at 4.0 μM. Piperine concentration varied from 0 to 12 μM. Experiments were carried out at room temperature (298 K).

The sample was excited at 295 nm using a picosecond pulsed light emitting diode (LED), and fluorescence decay was collected using a 340 nm filter. The fluorescence decay profile (Figure S2) was fitted using multiexponential decay (Equation (9)), where τ_i_ is the lifetime of each component, and α_i_ is the contribution of each component to total fluorescence decay. The average lifetime <τ_avg_> was calculated using Equation (10) (Table S1).

(9) $$I_{T}=\overset{n}{\underset{i=1}{\sum }}\alpha_{i}\cdot e^{\frac{-T}{\tau_{i}}}$$

(10) $$\tau_{avg}=\frac{{\alpha_{1}\tau_{1}}^{2}+{\alpha_{2}\tau_{2}}^{2}}{\alpha_{1}\tau_{1}+\alpha_{2}\tau_{2}}$$

### 3.4. Circular Dichroism Spectroscopy

Circular dichroism spectra were recorded at 288 K, 298 K, and 308 K on a Jasco J-710 spectropolarimeter model DRC-H (Jasco, Easton, MD, USA) equipped with a demountable quartz cell with a 0.01 cm optical path length. The CD spectra were recorded from the 200 nm to 260 nm range with a scan rate of 20 nm/min and a spectral resolution of 0.1 nm. For each spectrum, 15 accumulations were performed. The molar ratios of RSA and piperine were 1:0 and 1:6, and the buffer spectrum was subtracted. The influence of methanol in the sample was tested with 1.2% of methanol in the solution. The ellipticity θ collected in millidegrees was converted to mean residue ellipticity [θ] (deg.cm^2^.dmol^−1^) using Equation (11).

(11) $$\left[\theta \right]=\frac{\theta \left(mdeg\right)}{10\cdot \left[P\right]\cdot l\cdot n}$$

### 3.5. Piperine Optimization by Ab Initio Calculations

The molecular piperine structure was optimized by ab initio calculation. The calculations were performed using the Gamess2013 [43,44] quantum mechanics package with Hartree–Fock (HF) formalism [45] and functional density theory (DFT) [46]. Then, 6-31+G (d, p) was used as the first set of bases followed by a structure refinement with the set of bases 6-311+G(2d,2p) and B3LYP functional [47]. The optimized alkaloid geometry was determined with a Polarizable Continuum Model (PCM) solvent model [48] for water with total charge as 0 and multiplicity as 1. The optimizations were followed by harmonic frequency calculations to obtain the vibrational, the rotational, and the translational contributions to the free energy. The Raman spectrum was calculated with a set of bases 6-31+G (d,p), functional B3LYP, and the Hessian matrix previously calculated in the structure optimization step. The electrostatic potential map and the partial charges were determined using the geodesic method [49] along with the same functional base sets and solvent model used for the optimization of the structure. The structure and the normal frequency modes analyses were visualized by wxMacMolPlt software [50]. The Raman spectrum was visualized by SciDavis (Free Software Foundation, Boston, MA, USA). These calculations were performed using the computational structure of GRID UNESP.

### 3.6. RSA Modeling

The comparative model for the RSA structure was built using MODELLER [51,52] with sequence–sequence, sequence–profile, and PSI-Blast as fold assignment methods. Equine serum albumin (5HOZ), which has 73% similarity with RSA, was used as a template. The stereochemical quality of the model was evaluated based on the Ramachandran plot, which was calculated by PROCHECK [53]. Then, the RSA structure was relaxed during 50 ns of molecular dynamics following the same methodology as described in Section 3.8.

### 3.7. Molecular Docking

The alkaloid structure and the partial charge used in molecular docking were obtained from ab initio calculations using Gamess2013(Ames laboratory, Ames, IA, USA). The AutoDockTools [54] software of the MGL program Tools 1.5.4 was used to prepare the RSA by adding polar hydrogen atoms and Gasteiger charges. The maps were generated by the AutoGrid 4.2 program [54] with a spacing of 0.375 Å, a dimension of 66 × 70 × 58 points, and grid center coordinates of 67.211, 105.553, and 50.661 for x, y, and z coordinates, respectively. The AutoDock 4.2 program [54] was used to investigate the RSA binding sites using the Lamarckian Genetic Algorithm (LGA) with a population size of 150, a maximum number of generations of 27,000, and energy evaluations equal to 2.5 × 10^6^. The other parameters were selected as software defaults. To generate different conformations, the total number of runs was set to 100. The final conformations were visualized by VMD [55].

### 3.8. Molecular Dynamics

The initial coordinates of the protein–ligand complexes were obtained by molecular docking. The GROMOS96/53a force field [56] was utilized as the force field, and molecular dynamics simulations were performed by GROMACS/5.1.4 [57]. The system was solvated with the Simple Point Charge (SPC) water model in a dodecahedral box, where the protein was centered and was at least 1 nm from the edge of the box. The system was neutralized with Na^+^ and Cl^−^ in a concentration of 150 mM. Energy minimization was performed with the steepest descent algorithm with 5000 steps and a tolerance of 10 kJ/mol. The first stage of equilibration, performed in NVT ensemble, took 100 ps of simulation at a constant temperature of 300 K (coupled to the modified Barendsen thermostat). Random velocities were generated by the Maxwell–Boltzman distribution. The second stage of equilibration, performed in the NPT ensemble, took 100 ps of simulation at a constant temperature of 300 K and a pressure of 1 atm (coupled to the Parrinello–Rahman barostat). The molecular dynamics simulations were performed with steps of 2 fs using the leap-frog algorithm to integrate the equations of motion. The final results were an average of three simulations. Molecular dynamics simulations were performed in GRID UNESP.

## 4. Conclusions

In the present work, a molecular investigation of the interaction between piperine and RSA was carried out by means of experimental and computational approaches. Steady-state and time-resolved fluorescence proved that the quenching mechanism was static. Three equivalent and independent binding sites with moderate binding constants were disclosed. The importance of this information lies in the fact that the investigated protein (RSA) has the biological characteristic of the transport of exogenous ligands. The thermodynamic parameters indicated that the complexation was a spontaneous process (ΔG = −25 kJ/mol), with the main contribution coming from the enthalpic term, which led to the conclusion that hydrogen bonds were fundamental to maintain the stability of the complex.

The model of the tertiary structure for RSA was built using equine serum albumin as a template, which reached 73% similarity. The RSA modeled presented high stereochemical quality, as shown by the Ramachandran plot. The molecular dynamics during 50 ns allowed the protein to relax in solution, and the percentage of secondary structures calculated matched the circular dichroism results.

Molecular docking results disclosed the microenvironment of the three binding sites with the presence of hydrogen bonds and polar residues in every site. These results were in agreement with the enthalpic character previously found by the Van’t Hoff analysis. The stability of the complex RSA–piperine at each site was verified with molecular dynamics during 50 ns, following the distance between both COG of RSA and piperine, which fluctuated around 1 nm, 0.9 nm, and 0.5 nm for sites 1, 2, and 3, respectively.

In conclusion, the multispectroscopical and the computational approaches elucidated, in detail, the piperine–RSA molecular interaction and its importance in supporting related branches of pharmacology, such as drug design and pharmacokinetics.

## Figures and Tables

**Table 1:** Values of the Stern–Volmer constant (K_SV_) for tryptophan fluorescence quenching of RSA, binding constants (K_a_), and the number of binding sites (n) for the interaction between RSA and piperine at 288 K, 298 K, and 308 K.

Temperature (K)	K_SV_ (×10^4^ M^−1^)	K_a_ (×10^4^ M^−1^)	n
**288**	3.77 ± 0.09	3.90 ± 0.67	0.93
**298**	2.78 ± 0.12	2.77 ± 0.54	0.88
**308**	2.54 ± 0.09	2.06 ± 0.65	0.83

**Table 2:** Thermodynamic parameters of the complex RSA–piperine at 288 K, 298 K, and 308 K.

T (K)	∆G (kJ/mol)	∆H (kJ/mol)	∆S (J/mol.K)	T. ∆S (kJ/mol)
**288**	−25.29 ± 0.95	−23.54 ± 0.52	6.1 ± 1.5	1.76 ± 0.43
**298**	−25.35 ± 0.96			1.82 ± 0.44
**308**	−25.41 ± 0.98			1.88 ± 0.46

**Table 3:** Secondary structures percentage of RSA obtained through circular dichroism (CD) of the RSA model obtained through comparative modeling and of the RSA model after 50 ns molecular dynamics (MD).

Methods	α-Helix (%)	Turns (%)	Coil (%)
**Circular Dichroism**	63	17	17
**RSA model**	75	11	11
**RSA model with MD**	65	17	16

## Supplementary Materials

Supplementary materials can be found at https://www.mdpi.com/1422-0067/20/12/2856/s1.

## Abbreviations

RSA	Rat Serum Albumin
PDB	Protein Data Bank
IDF	Interaction Density Function
CD	Circular Dichroism
λ_exc_	Excitation wavelenght
ΔH	Enthalpy variation
ΔS	Entropy variation
ΔG	Gibbs free energy
MEP	Potential electrostatic map
MD	Molecular Dynamics
VMD	Visual Molecular Dynamics software
