# Supplementary material for: Experimental Approaches and Computational Modeling of Rat Serum Albumin and Its Interaction with Piperine
Source: Int J Mol Sci. 2019 Jun 12;20(12):2856. doi: 10.3390/ijms20122856 (PMC6627779; doi:10.3390/ijms20122856)
Supplement: Supplementary file 1 [file ijms-20-02856-s001.pdf]

International Journal of *Molecular Sciences*

Supplementary material

Experimental Approaches and Computational Modeling of Rat Serum Albumin and Its Interaction with Piperine

Gabriel Zazeri [1], Ana Paula Ribeiro Povinelli [1], Marcelo de Freitas Lima [2] and Marinônio Lopes Cornélio [1,*]

1 Departamento de Física, Instituto de Biociências, Letras e Ciências Exatas (IBILCE), UNESP, Rua Cristovão Colombo 2265, CEP 15054-000, São José do Rio Preto, SP, Brazil; gabriel.zazeri@unesp.br (G.Z.), ana.povinelli@unesp.br (A.P.R.P.)
2 Departamento de Química, Instituto de Biociências, Letras e Ciências Exatas (IBILCE), UNESP, Rua Cristovão Colombo 2265, CEP 15054-000, São José do Rio Preto, SP, Brazil. marcelo.f.lima@unesp.br
* Correspondence: m.cornelio@unesp.br

Figure S1 shows that methanol did not influenced RSA fluorescence signal. In this way, the fluorescence quenching observed when piperine was added to the system was due to the molecule.

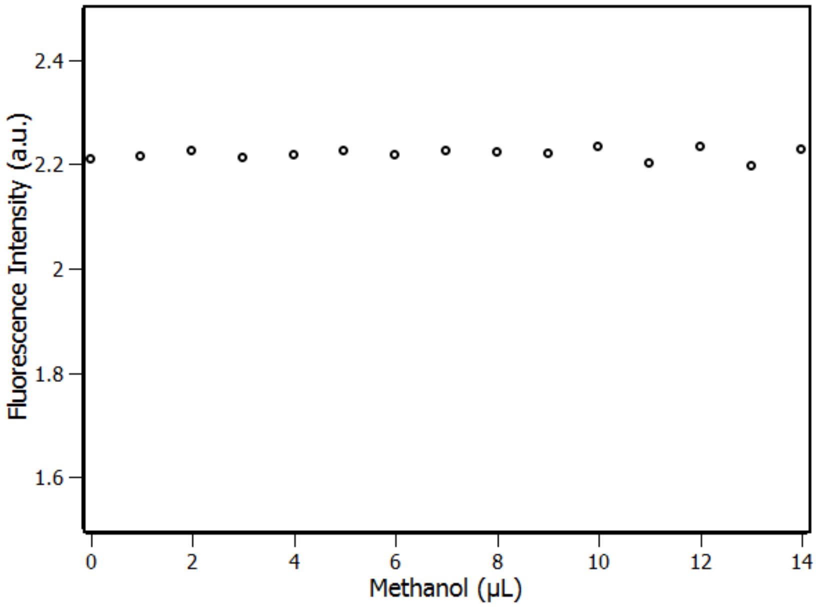

Figure 1. RSA fluorescence intensity at 340nm varying methanol concentrations. [RSA]=4μM, [methanol]:0 - 14μL. In these experiments temperature remained at 288K, and RSA was excited at 295nm.

Figure S2 shows that piperine did not have strong influence in the fluorescence decay.

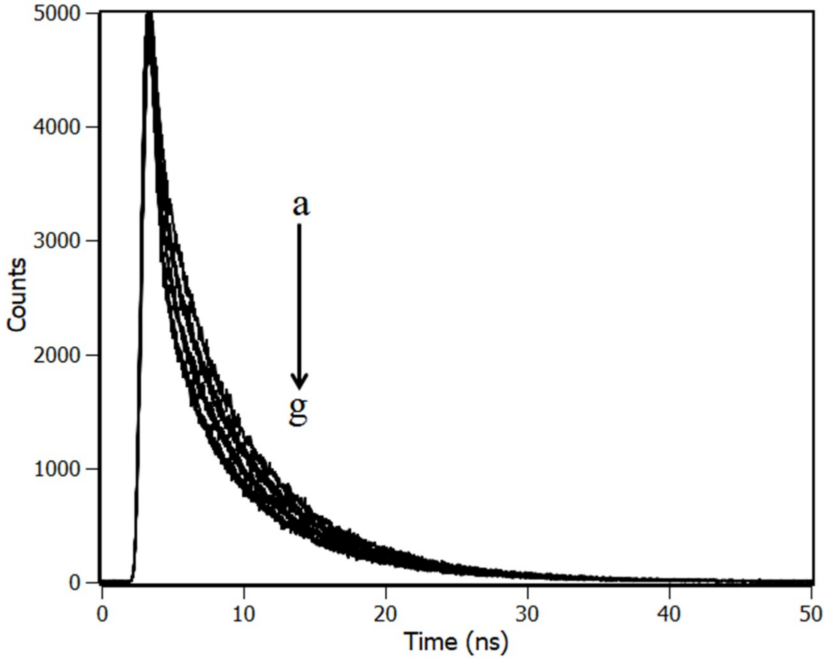

Figure S1: Time-dependent fluorescence decay of (a) RSA and (→ g) in the RSA:Piperine stoichiometries 1:0.5, 1:1, 1:1.5, 1:2, 1:2.5 and 1:3.

Time-dependent fluorescence decay was fitted using multiexponencial decay (Equation 10). The best fit was obtained with two lifetimes τ_1 and τ_2 (Table S1). The average time (τ_{avg}) was calculated by Equation 11 considering the contributions, α_1 and α_2, of each component.

Table S1: Tryptophan lifetime in different stoichiometries RSA:Piperine.

RSA : Piperine	α_1	τ_1 (ns)	α_2	τ_2 (ns)	τ_{avg} (ns)
**1:0**	0.09	1.20	0.91	6.67	6.43
**1:0.5**	0.09	1.13	0.91	6.50	6.41
**1:1**	0.1	1.16	0.9	6.48	6.37
**1:1.5**	0.11	1.07	0.89	6.43	6.32
**1:2**	0.12	0.98	0.88	6.35	6.24
**1:2.5**	0.12	0.88	0.88	6.28	6.17
**1:3**	0.14	0.92	0.86	6.29	6.16

The stability of RSA secondary structures at 288, 298 and 308K was verified by circular dichroism experiments (Figure S3 and Table S2).

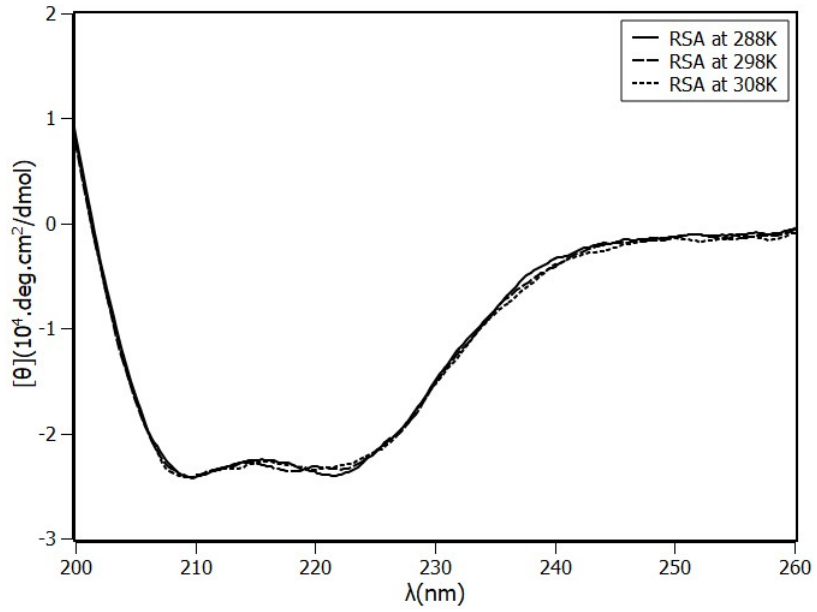

Figure S2: Circular Dichroism of RSA at 288K, 298K and 308K. [RSA]=4μM

Circular dichroism experiments also showed that piperine did not cause major structural change in RSA (Figure S4 and Table S2).

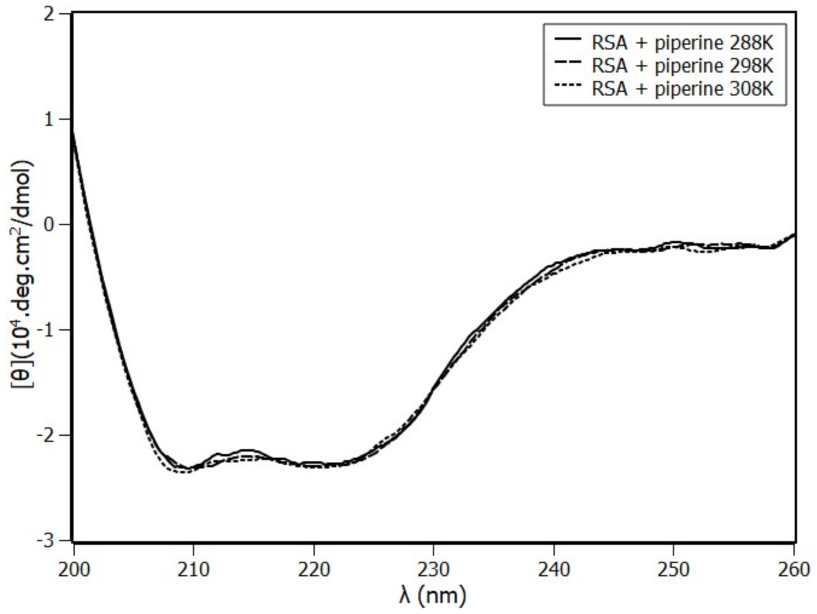

Figure S3: Circular Dichroism of RSA with piperine (1:6) and 1.2% of methanol at 288K, 298K and 308K. [RSA]=4μM.

Table 2. Main composition of secondary structures of RSA pure at 288K, 298K and 308K. And RSA:Piperine (1:6) with 1.2% at 288K, 298K and 308K.

Amostra	α-helix	Turns	Random Coil
**RSA 288K**	63%	17%	16%
**RSA 298K**	63%	17%	17%
**RSA 308K**	62%	17%	19%
**RSA+Pip 288K**	63%	20%	12%
**RSA+Pip 298K**	63%	20%	14%
**RSA+Pip 308K**	60%	20%	17%

Figure S4 presents the sequence alignment of RSA and ESA (PDB: 5HOZ). The sequences present 73% of similarity, which makes the use of comparative modeling possible.

```
RSA            1   EAHKSEIAHRFKDLGEQHFKGLVLIAFSQYLQKCPYEEHIKLVQEVTDFAKTCVADENAE    60
ESA(5HOZ)      1   DTHKSEIAHRFNDLGEKHFKGLVLVAFSQYLQQCPFEDHVKLVNEVTEFAKKCAADESAE    60
                   ::*********:****:*******:*******:**:*:*:***:***:***.*.***.**

RSA           61   NCDKSIHTLFGDKLCAIPKLRDNYGELADCCAKQEPERNECFLQHKDDNPNLPPFQRPEA   120
ESA(5HOZ)     61   NCDKSLHTLFGDKLCTVATLRATYGELADCCEKQEPERNECFLTHKDDHPNLPKL-KPEP   119
                   *****:*********:: .** .******** *********** ****:**** : :**

RSA          121   EAMCTSFQENPTSFLGHYLHEVARRHPYFYAPELLYYAEKYNEVLTQCCTESDKAACLTP   180
ESA(5HOZ)    120   DAQCAAFQEDPDKFLGKYLYEVARRHPYFYGPELLFHAEEYKADFTECCPADDKLACLIP   179
                   :* *::***:* .***:**:**********.****::**:*:  :*:**  .** *** *

RSA          181   KLDAVKEKALVAAVRQRMKCSSMQRFGERAFKAWAVARMSQRFPNAEFAEITKLATDVTK   240
ESA(5HOZ)    180   KLDALKERILLSSAKERLKCSSFQNFGERAVKAWSVARLSQKFPKADFAEVSKIVTDLTK   239
                   ****:**: *:::.::*:****:*.*****.***:***:**:**:*:***::*:.**:**

RSA          241   INKECCHGDLLECADDRAELAKYMCENQATISSKLQACCDKPVLQKSQCLAEIEHDNIPA   300
ESA(5HOZ)    240   VHKECCHGDLLECADDRADLAKYICEHQDSISGKLKACCDKPLLQKSHCIAEVKEDDLPS   299
                   ::****************:****:**:* :**.**:******:****:*:**::.*::*:

RSA          301   DLPSIAADFVEDKEVCKNYAEAKDVFLGTFLYEYSRRHPDYSVSLLLRLAKKYEATLEKC   360
ESA(5HOZ)    300   DLPALAADFAEDKEICKHYKDAKDVFLGTFLYEYSRRHPDYSVSLLLRIAKTYEATLEKC   359
                   ***::****.****:**:* :***************************:**.********

RSA          361   CAEGDPPACYGTVLAEFQPLVEEPKNLVKTNCELYEKLGEYGFQNAVLVRYTQKAPQVST   420
ESA(5HOZ)    360   CAEADPPACYRTVFDQFTPLVEEPKSLVKKNCDLFEEVGEYDFQNALIVRYTKKAPQVST   419
                   ***.****** **: :* *******.***.**:*:*::***.****::****:*******

RSA          421   PTLVEAARNLGRVGTKCCTLPEAQRLPCVEDYLSAILNRLCVLHEKTPVSEKVTKCCSGS   480
ESA(5HOZ)    420   PTLVEIGRTLGKVGSRCCKLPESERLPCSENHLALALNRLCVLHEKTPVSEKITKCCTDS   479
                   ***** .*.**:**::**.***::**** *::*:  ****************:****:.*

RSA          481   LVERRPCFSALTVDETYVPKEFKAETFTFHSDICTLPDKEKQIKKQTALAELVKHKPKAT   540
ESA(5HOZ)    480   LAERRPCFSALELDEGYVPKEFKAETFTFHADICTLPEDEKQIKKQSALAELVKHKPKAT   539
                   *.********* :** **************:******:.*******:*************

RSA          541   EDQLKTVMGDFAQFVDKCCKAADKDNCFATEGPNLVARSKEALA                   584
ESA(5HOZ)    540   KEQLKTVLGNFSAFVAKCCGAEDKEACFAEEGPKLVASSQLALA                   583
                   ::*****:*:*: ** *** * **: *** ***:*** *: ***
```

Figure S5: Rat serum albumin (RSA) and Equine serum albumin (ESA) sequence alignment with 73% of similarity.

The “PDBQT file of optimized piperine structure” shows the position of each atom in the optimized piperine structure and their respective charges. The structures and charges presented in this file were used in molecular docking and molecular dynamic calculations.

PDBQT file of optimized piperine structure

```
REMARK  0 active torsions:
REMARK  status: ('A' for Active; 'I' for Inactive)
REMARK     I    between atoms: C_1  and  C_9
REMARK     I    between atoms: C_2  and  C_12
REMARK     I    between atoms: C_13  and  C_14
REMARK     I    between atoms: C_14  and  N_16
ROOT
HETATM   1  C   LIG    1      8.391   2.457   2.690  0.00  0.00    -0.182 C
HETATM   2  C   LIG    1      9.341   1.820   3.405  0.00  0.00    -0.203 C
HETATM   3  C   LIG    1      2.986   2.020   0.268  0.00  0.00     0.272 C
HETATM   4  O   LIG    1      4.153   2.854   0.205  0.00  0.00    -0.411 OA
HETATM   5  O   LIG    1      3.388   0.783   0.883  0.00  0.00    -0.405 OA
HETATM   6  C   LIG    1      5.074   2.272   1.039  0.00  0.00     0.378 A
HETATM   7  C   LIG    1      4.613   1.027   1.442  0.00  0.00     0.321 A
HETATM   8  C   LIG    1      6.295   2.758   1.434  0.00  0.00    -0.468 A
HETATM   9  C   LIG    1      7.091   1.944   2.275  0.00  0.00     0.183 A
HETATM  10  C   LIG    1      6.607   0.688   2.671  0.00  0.00    -0.238 A
HETATM  11  C   LIG    1      5.361   0.209   2.261  0.00  0.00    -0.341 A
HETATM  12  C   LIG    1     10.597   2.431   3.748  0.00  0.00     0.063 C
HETATM  13  C   LIG    1     11.581   1.813   4.428  0.00  0.00    -0.467 C
HETATM  14  C   LIG    1     12.840   2.515   4.769  0.00  0.00     0.664 C
HETATM  15  O   LIG    1     12.923   3.750   4.656  0.00  0.00    -0.699 OA
ENDROOT
BRANCH  14  16
HETATM  16  N   LIG    1     13.884   1.775   5.241  0.00  0.00    -0.238 N
HETATM  17  C   LIG    1     15.104   2.443   5.704  0.00  0.00     0.035 C
HETATM  18  C   LIG    1     16.311   2.062   4.844  0.00  0.00     0.058 C
HETATM  19  C   LIG    1     16.481   0.542   4.780  0.00  0.00    -0.032 C
HETATM  20  C   LIG    1     15.175  -0.131   4.347  0.00  0.00     0.088 C
HETATM  21  C   LIG    1     13.996   0.314   5.223  0.00  0.00    -0.053 C
ENDBRANCH  14  16
TORSDOF 0
```

Figure S6 shows the theoretic Raman Spectrum calculated after the optimization of piperine by *ab initio* methods.

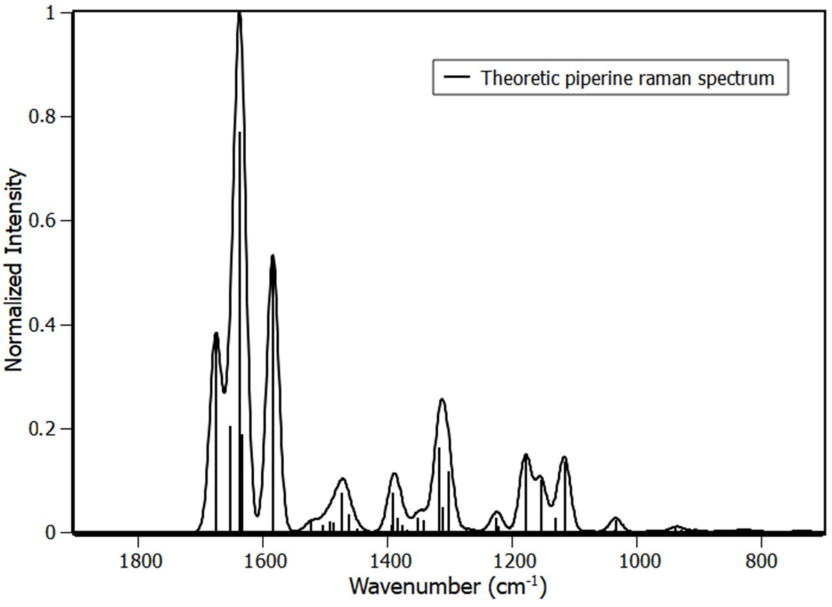

Figure S7: Piperine Raman spectrum calculated by *ab initio* methods.

Figures below present the stability of RSA during the 50ns of molecular dynamics when piperine was in different binding sites. The root mean square deviation (RMSD) and the radius of gyration (RG) revealed small structural fluctuations for RSA, regardless the site where piperine is in. The stability of the complex RSA-Piperine was verified by the distance from the center of geometry (COG) of piperine to COG of RSA.

Piperine in site 1

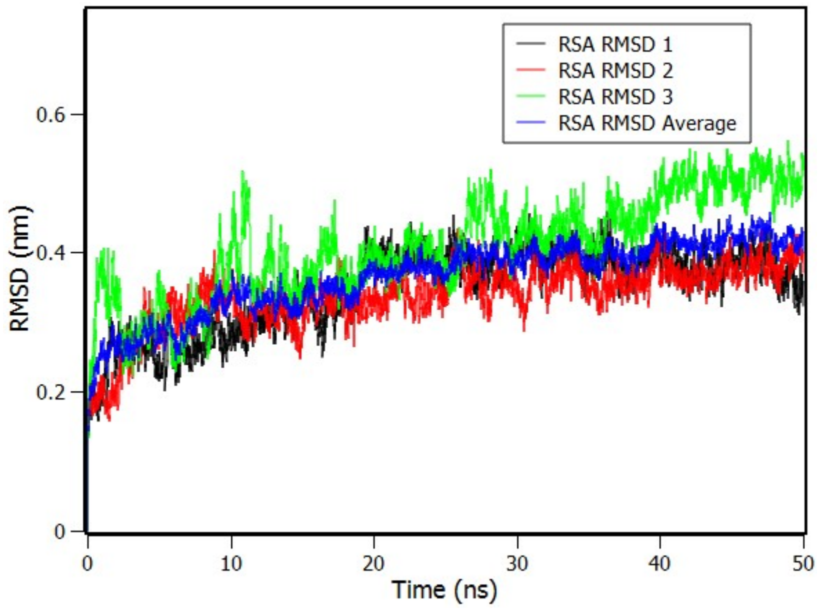

Figure S7: RMSD of RSA calculated from three simulations with piperine in Site 1 (RSA RMSD 1, 2 and 3), and the average of them (RSA RMSD Average).

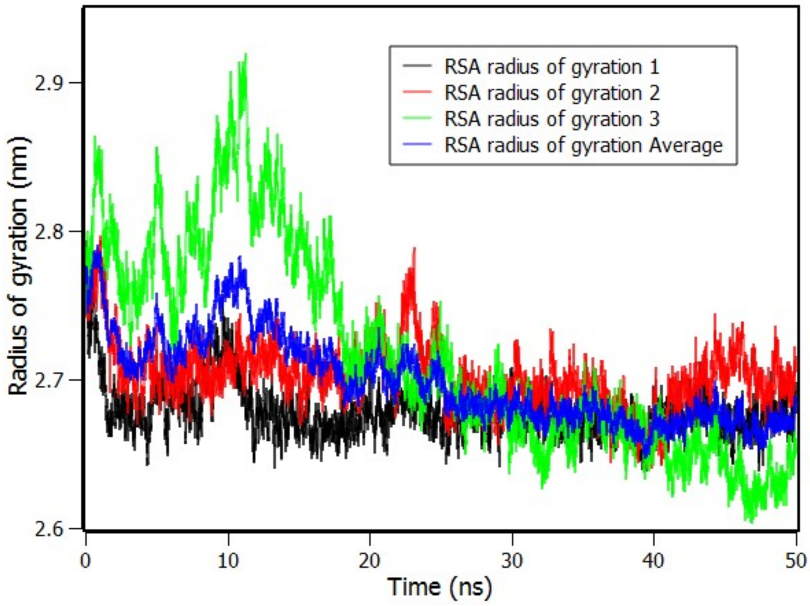

Figure S8: RG of RSA calculated from three simulations with piperine in Site 1 (RSA radius of gyration 1, 2 and 3), and the average of them (RSA radius of gyration Average).

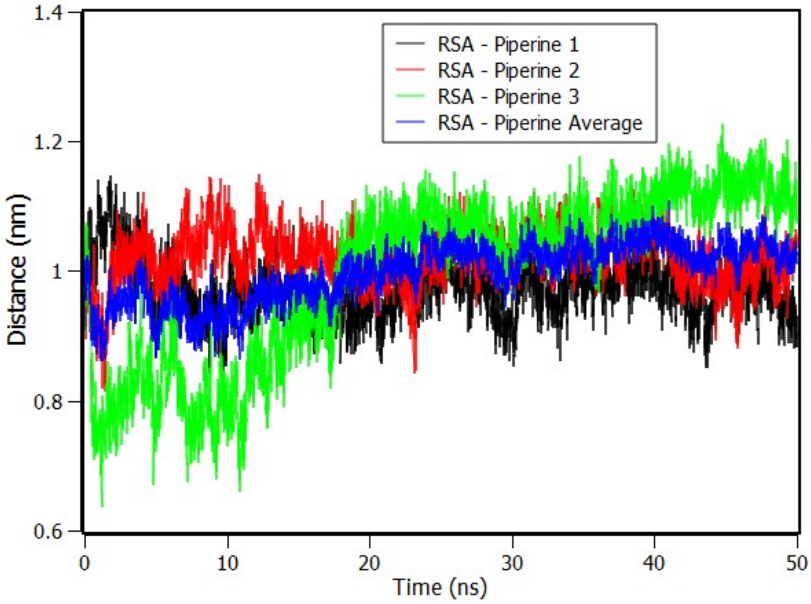

Figure S9: Distance from COG of RSA to COG of piperine (in site 1) calculated from three simulations (RSA-Piperine 1, 2 and 3) and the average of them (RSA-Piperine Average).

Piperine in site 2

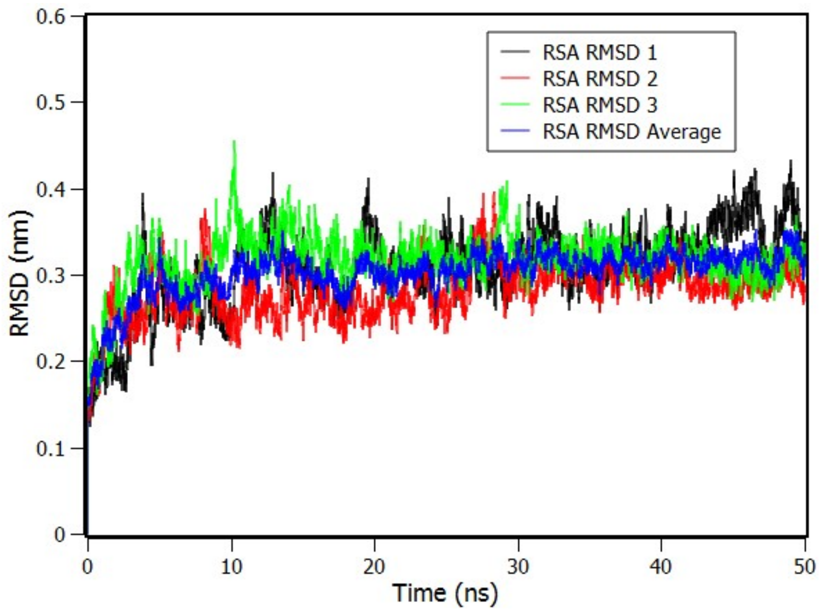

Figure S10: RMSD of RSA calculated from three simulations with piperine in Site 2 (RSA RMSD 1, 2 and 3), and the average of them (RSA RMSD Average).

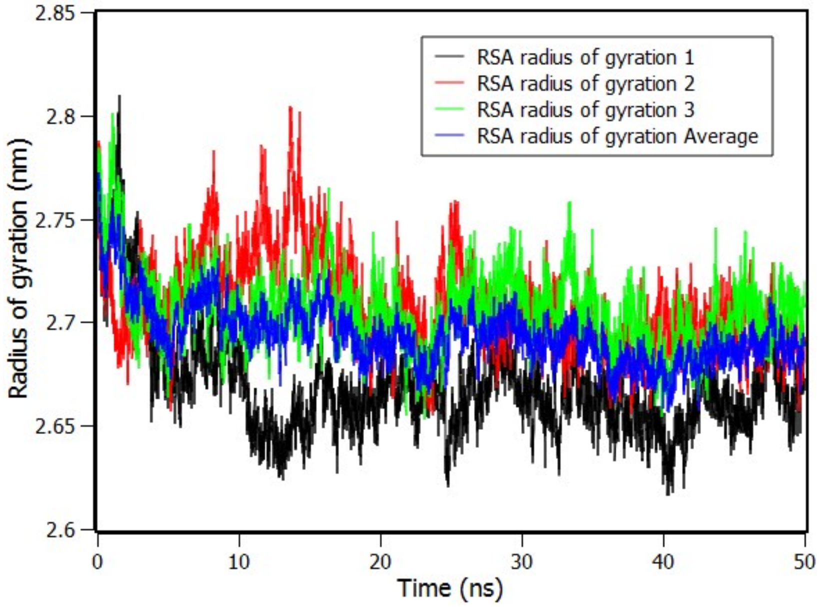

Figure S9: RG of RSA calculated from three simulations with piperine in Site 2 (RSA radius of gyration 1, 2 and 3), and the average of them (RSA radius of gyration Average).

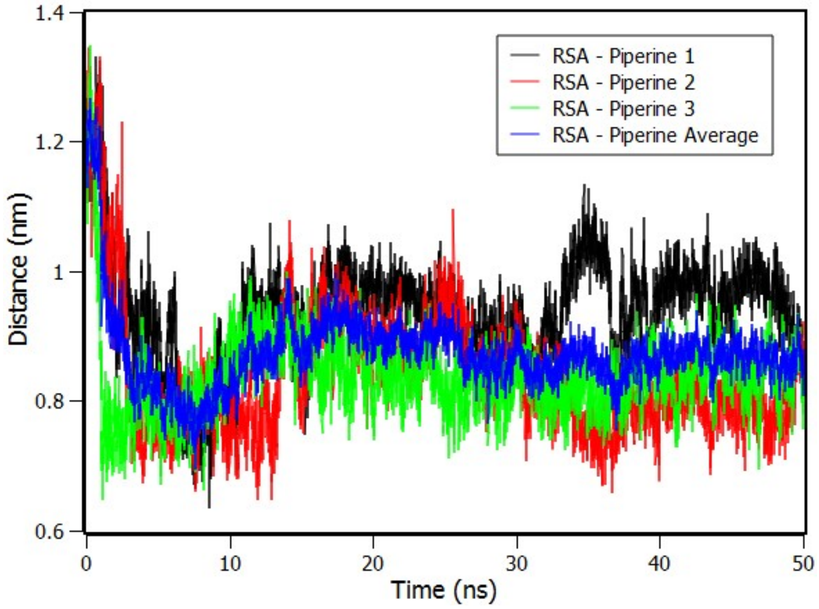

Figure S102: Distance from COG of RSA to COG of piperine (in site 2) calculated from three simulations (RSA-Piperine 1, 2 and 3) and the average of them (RSA-Piperine Average).

Piperine in site 3

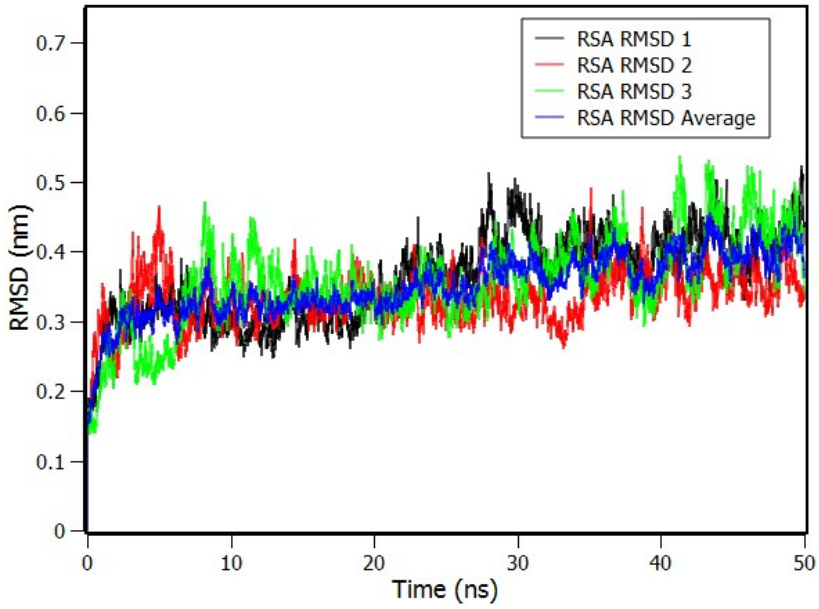

Figure S11: RMSD of RSA calculated from three simulations with piperine in Site 3 (RSA RMSD 1, 2 and 3), and the average of them (RSA RMSD Average).

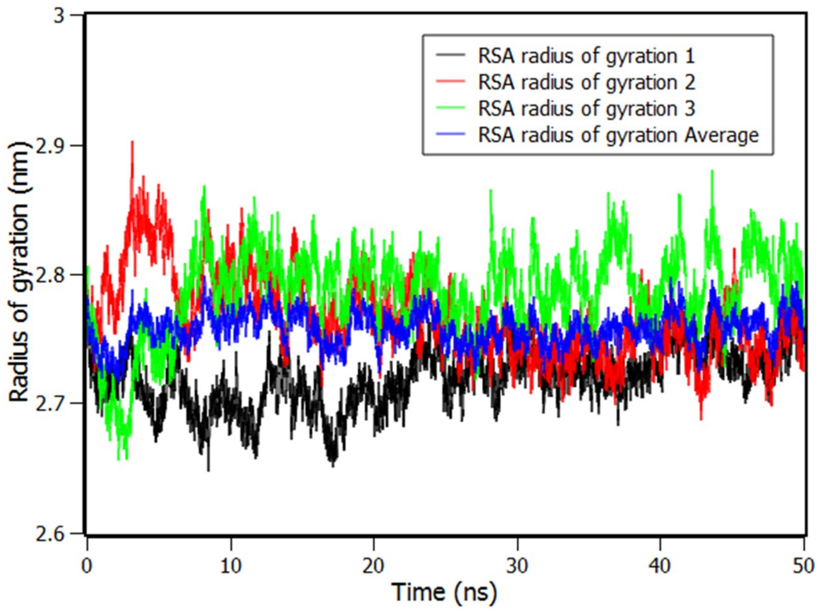

Figure S12: RG of RSA calculated from three simulations with piperine in Site 3 (RSA radius of gyration 1, 2 and 3), and the average of them (RSA radius of gyration Average).

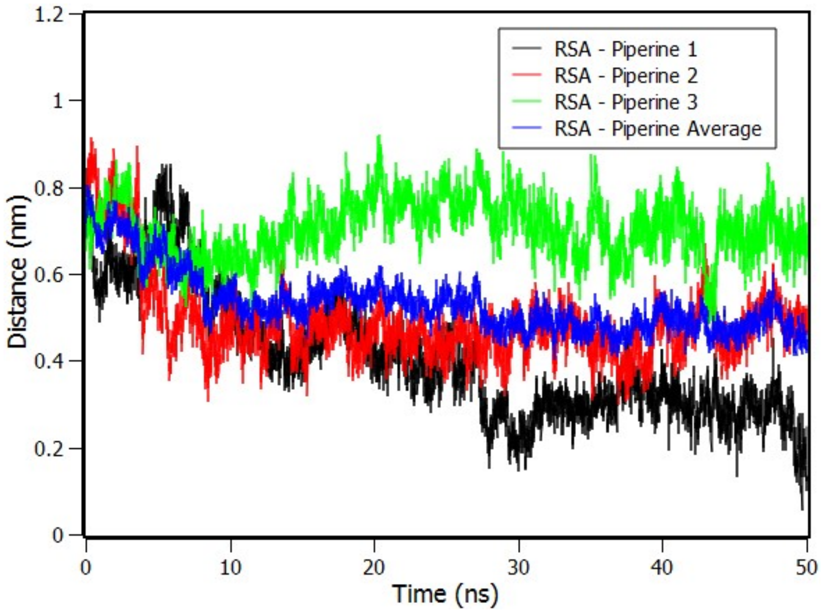

Figure S13: Distance from COG of RSA to COG of piperine (in site 3) calculated from three simulations (RSA-Piperine 1, 2 and 3) and the average of them (RSA-Piperine Average).
